# Supplementary figures and images for: The LINC Complex Inhibits Excessive Chromatin Repression
Source: Cells. 2023 Mar 18;12(6):932. doi: 10.3390/cells12060932 (PMC10047284; doi:10.3390/cells12060932)

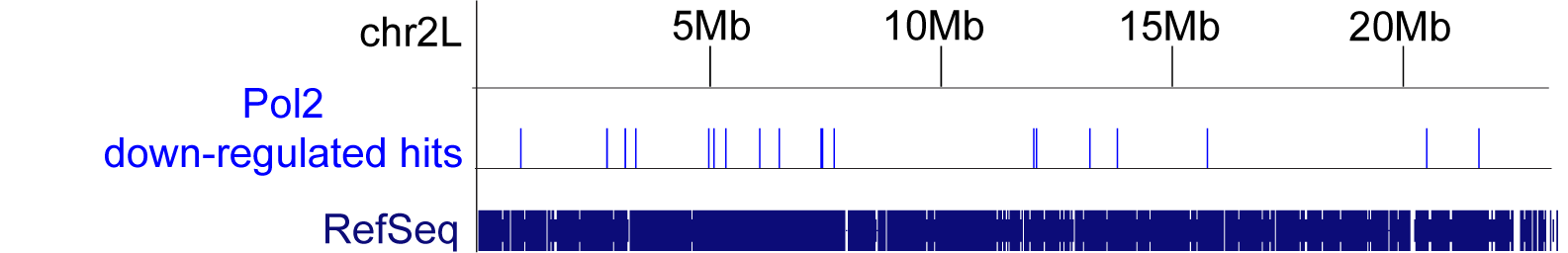

Supplement: Supplementary file 1 [file cells-12-00932-s001.zip › Supp Figure S1 .tif]

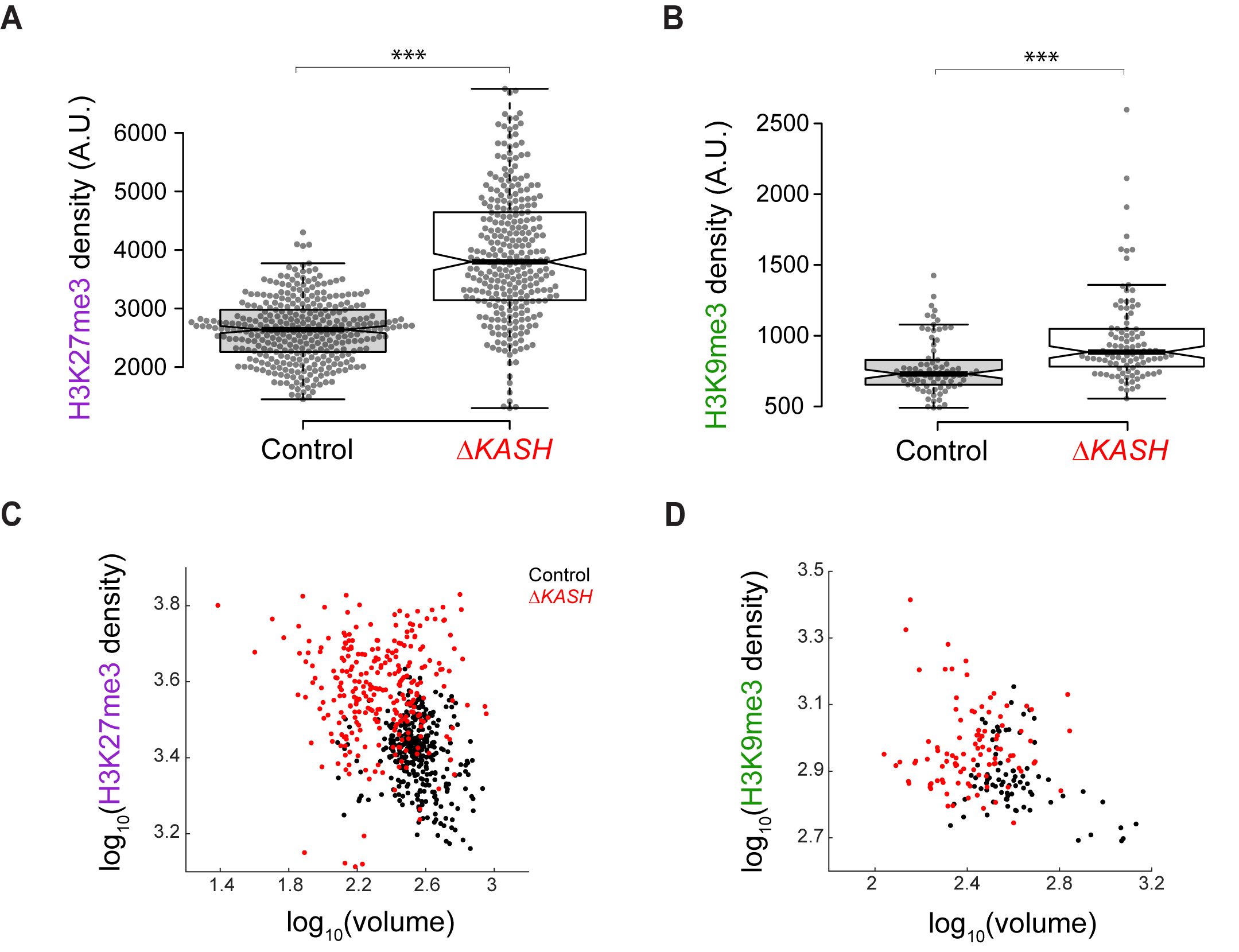

Supplement: Supplementary file 1 [file cells-12-00932-s001.zip › Supp Figure S2.tif]

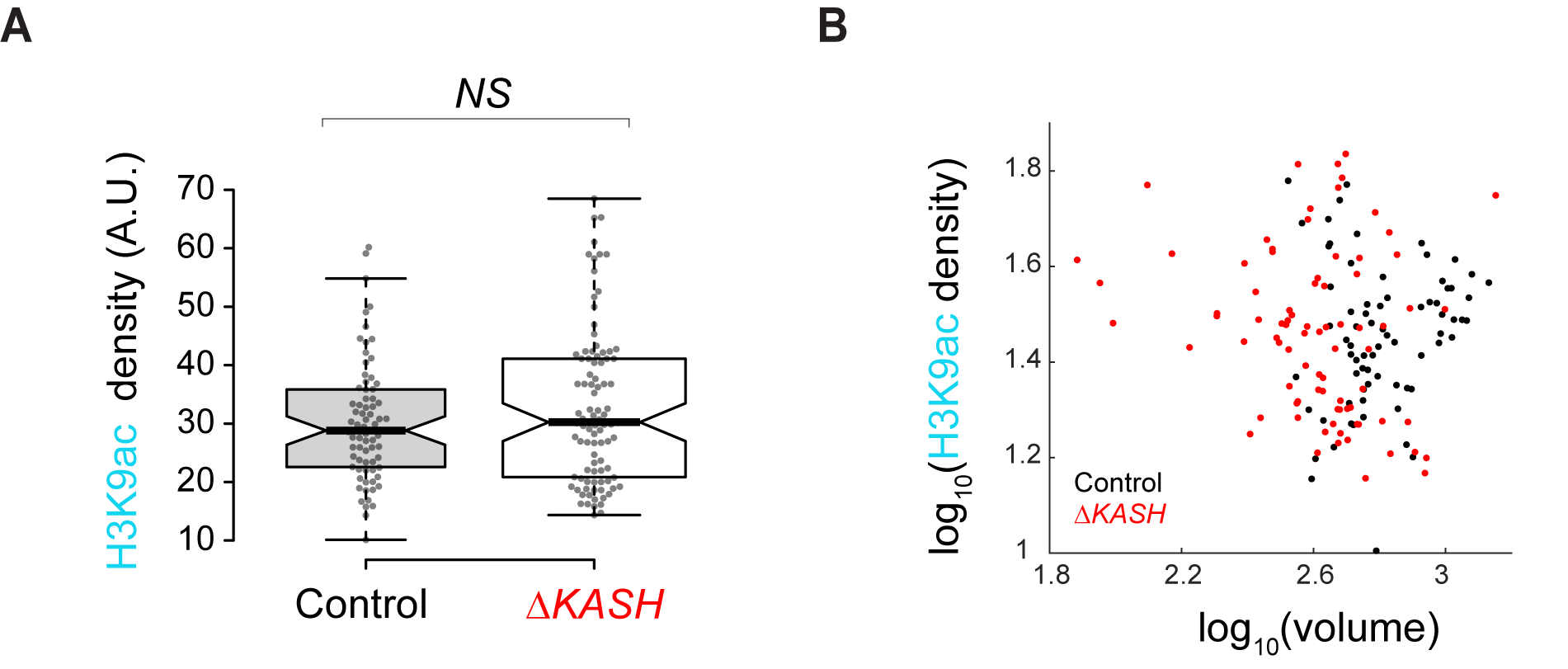

Supplement: Supplementary file 1 [file cells-12-00932-s001.zip › Supp Figure S3.tif]

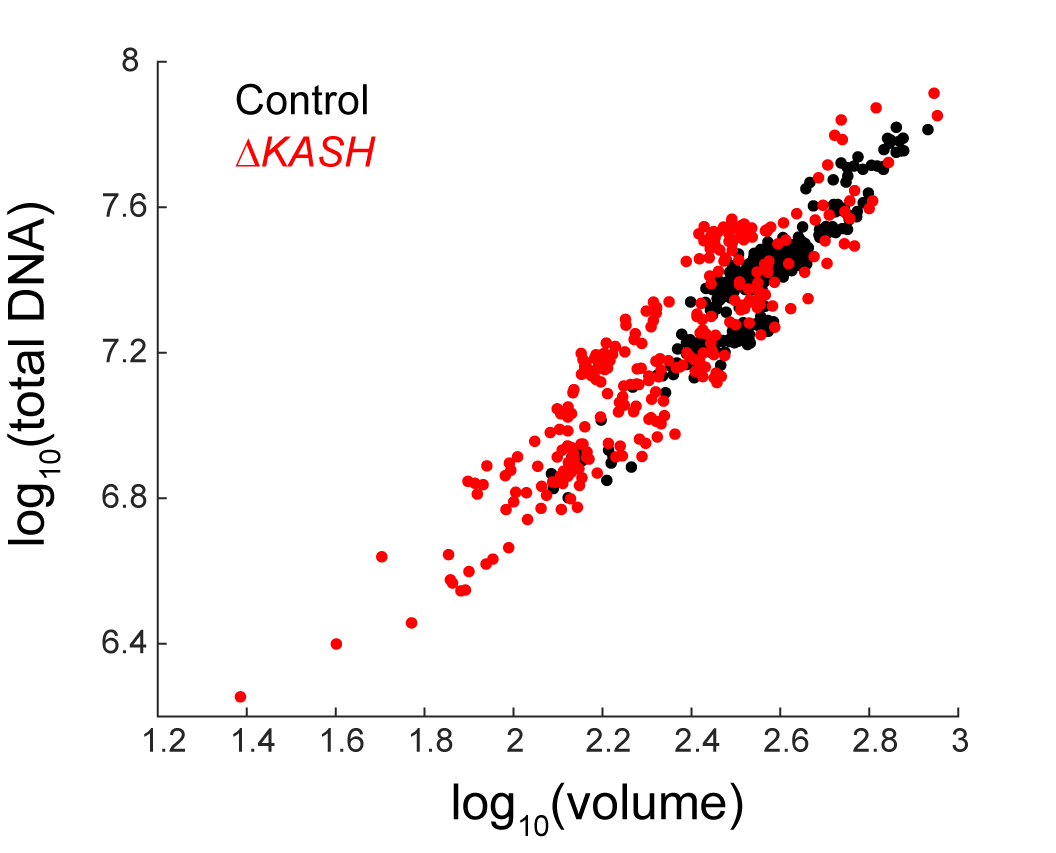

Supplement: Supplementary file 1 [file cells-12-00932-s001.zip › Supp Figure S4.tif]

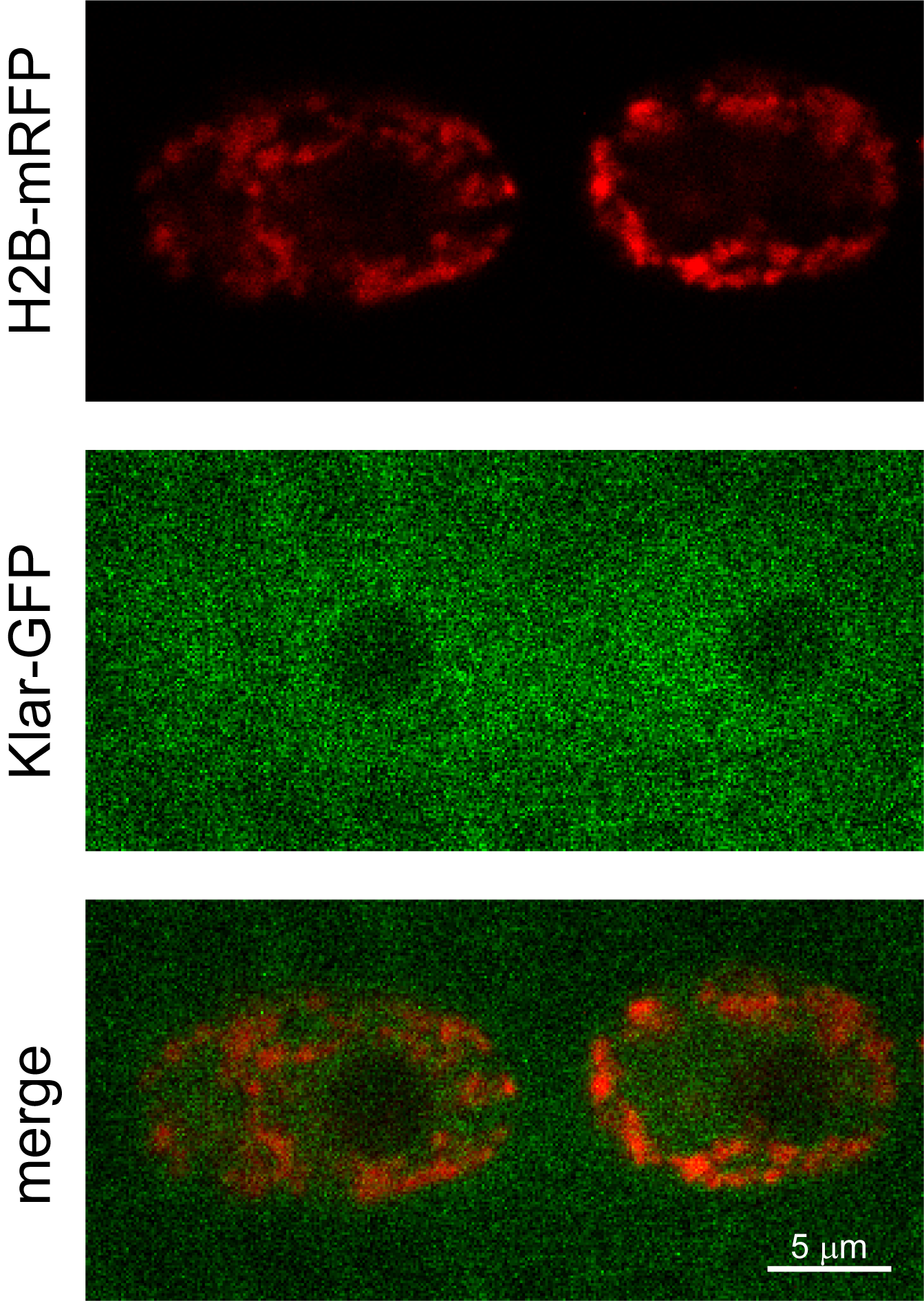

Supplement: Supplementary file 1 [file cells-12-00932-s001.zip › Supp Figure S5.tif]

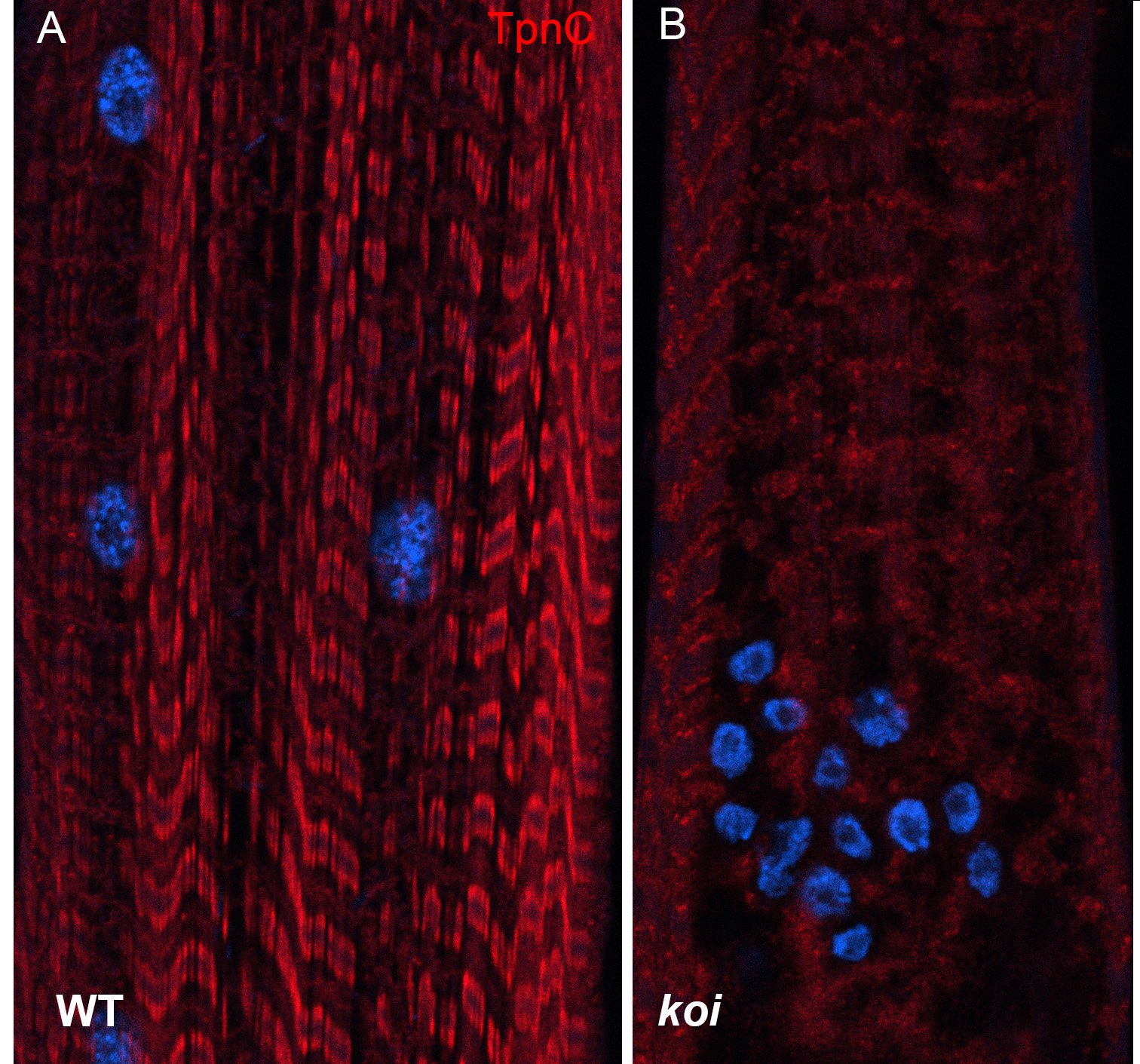

Supplement: Supplementary file 1 [file cells-12-00932-s001.zip › Supp Figure S6.jpg]
